# Critical Overview of Serous Endometrial Intraepithelial Cancer Treatment: Systematic Review of Adjuvant Options

**DOI:** 10.3390/life13071429

**Published:** 2023-06-22

**Authors:** Carlo Ronsini, Antonella Reino, Rossella Molitierno, Maria Giovanna Vastarella, Elvira La Mantia, Pasquale De Franciscis

**Affiliations:** 1 Department of Woman, Child and General and Specialized Surgery, University of Campania “Luigi Vanvitelli”, Largo Madonna Delle Grazie, 1, 80138 Naples, Italy; carlo.ronsini@unicampania.it (C.R.); mariagiovanna.vastarella@studenti.unicampania.it (M.G.V.); 2 Pathology Unit, University of Campania “L. Vanvitelli”, Via Luciano Armanni, 80138 Naples, Italy

**Keywords:** serous endometrial intraepithelial carcinoma, SEIC, MUSC, adjuvant therapy, relapse

## Abstract

SEIC is a non-invasive lesion of the endometrial epithelium considered to be the precursor to uterine serous carcinoma (USC) and is just as aggressive as USC. Currently, there are no reliable data about the behavior and prognosis of SEIC; therefore, the therapeutic management approach is not clear. **Method**: A systematic search of the Pubmed, Scopus and Embase databases was conducted, following the recommendations in the Preferred Reporting Items for Systematic Reviews and Meta-Analyses (PRISMA). **Results**: Of the 296 studies that matched the search criteria, only 9 met the inclusion criteria, covering a total of 81 patients. The main disease-presenting pattern was AUB (abnormal uterine bleeding). In 31 cases, SEIC was associated with extrauterine disease. All patients underwent hysterectomy and salpingo-oophorectomy, while only 15 of the 81 patients received adjuvant treatments. In the patients receiving adjuvant therapy, the RR was 42.67%, the DFS was 35.71% and the OS was 57.13%. In patients subjected to follow-up alone, the RR was only 28.78%, the DFS was 59.1% and the OS was 66.6%. **Conclusions**: The presence of an extrauterine disease significantly worsens outcomes, regardless of adjuvant treatment. In cases of disease confined to the uterine mucosa alone, the prognosis is good and follow-up allows a good control of the disease; however, adjuvant therapy could further increase survival rates and reduce relapse rates.

## 1. Introduction

Serous endometrial intraepithelial carcinoma (SEIC) is also described in the literature as ‘minimal uterine serous carcinoma’ (MUSC), ‘serous endometrial intraepithelial neoplasia’, ‘endometrial carcinoma in situ’, ‘non-invasive endometrial serous carcinoma’ and ‘superficial serous carcinoma’. It represents a non-invasive lesion of the endometrial epithelium [1]. As with invasive serous carcinoma of the uterus (USC), it constitutes an aggressive histological form of type II endometrial cancer. Although previously considered a pre-cursor of USC, recent evidence demonstrates its aggressive behavior and its ability to extensively metastasize extrauterine, despite the absence of myometrial invasion and lymph-vascular involvement [2,3]. Several studies suggest that superficial intraepithelial carcinoma may spread through the tubes or lymphatic vessels within the peritoneal cavity. However, its behavior is currently unpredictable [4]. Although there are no International guidelines, the WHO recommends surgical staging of the disease (hysterectomy, bilateral salpingo-oophorectomy, omentectomy, lymph node removal, or peritoneal dissection and biopsy). The scarcity with which it occurs and the variable prognosis reported in the literature mean that there is no non-ambiguous adjuvant therapy. Data are available in the literature on the use of chemotherapy and on follow-up models [4,5,6]. The purpose of this review is to investigate the effects of adjuvant treatments on the prognosis of the disease.

## 2. Materials and Methods

The selected registrations complied with the PRISMA (Preferred Reporting Items for Systematic Reviews and Meta-Analysis) guidelines [7]. We registered this systematic review on the PROSPERO site with protocol number 403507.

### 2.1. Search Methods 

A systematic search of the Pubmed, EMBASE and Scopus databases was carried out in February 2023. Studies were considered if they had been included in published material since their first release. No country restrictions were carried out. The search criteria adopted to identify studies applicable to the subject of the review were: “intraepithelial serum endometrial carcinoma” OR “minimal serous uterine carcinoma” OR “SEIC”.

### 2.2. Studies Selection 

**Figure 1:**
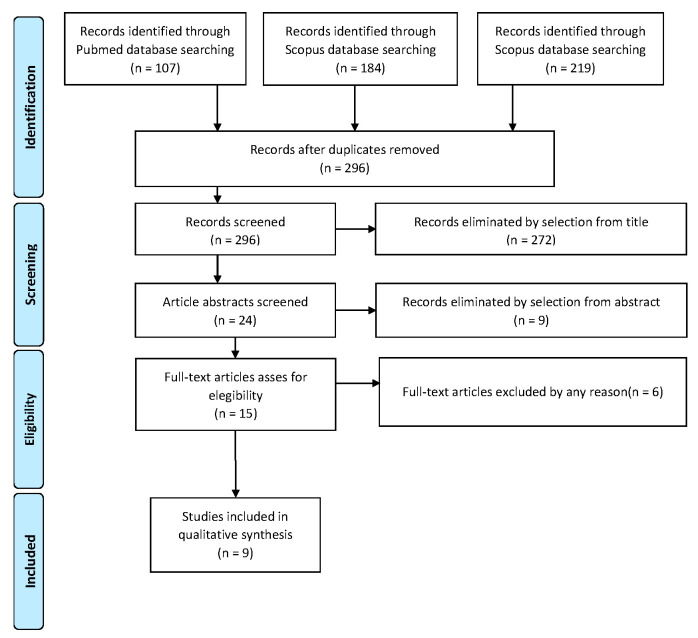
PRISMA Flow-chart.

Study selection was made independently by AR and RM. In disputed cases, CR decided to include or exclude. The inclusion criteria were: (1) studies including patients with the diagnosis of serous intraepithelial uterine carcinoma; (2) studies including patients who underwent surgical staging; (3) studies reporting at least one outcome of interest (recurrence rate; recurrence type; surgical staging; type of adjuvant treatment; survival report); and (4) peer-reviewed articles, published originally. Nonoriginal studies, preclinical trials, animal trials, abstract-only publications and articles in languages other than English were excluded. Wherever possible, the authors of studies that were only published as congress abstracts were contacted via email and asked to provide their data. The primary outcome of interest was the recurrence of the disease. The studies selected and all reasons for exclusion are given in the Preferred Reporting Items for Systematic Reviews and Meta-Analyses (PRISMA) flowchart (Figure 1). All included studies were assessed regarding potential conflicts of interest.

### 2.3. Data Extraction

AR and RM extracted data for all relevant series and case reports. We extracted data on tumor characteristics (stage, histological subtype, LVSI status, grading), surgical approach, morbidity and oncological issues such as recurrences, deaths and recurrence rate (RR). We also collected data on adjuvant therapy (Number of cycles, type of drugs). In addition, data on follow-up and survival status reported as “not evident disease (NED”, “alive with disease (AWD)”, “death of disease (DOD)” and “death of other causes (DOC)” were also extracted. We also evaluated disease-free survival (DFS) as the time in months from the surgery to the recurrence or the last follow-up and overall survival (OS) as the time in months from the diagnosis to the recurrence or the last follow-up.

### 2.4. Quality Assessment

We assessed the quality of the included studies using the Newcastle–Ottawa scale (NOS) [8]. This assessment scale uses three broad factors (selection, comparability and outcome), with the scores ranging from 0 (lowest quality) to 8 (best quality). Two authors (CR and PDF) independently rated the study’s quality. Any disagreement was subsequently resolved by discussion or consultation with AR. We report the NOS Scale scores in Appendix A.

## 3. Results

### 3.1. Studies’ Characteristics

After the database search, 296 articles matched the search criteria. After removing records with no full text, duplicates and wrong study design (e.g., reviews), 15 were eligible. Of those, nine matched the inclusion criteria and were included in the systematic review. All nine studies were non-comparative, single-armed, or case-report studies evaluating the therapeutic management of SEICs. The countries where the studies were conducted, the publication year range, the study design, the type of surgery and adjuvant therapy, the mean follow-up and the number of participants are summarized in Table 1. Overall, the publication years ranged from 2000 to 2021 while the follow-up period ranged from 9 to 84 months.

### 3.2. Patients’ Characteristics

The clinical characteristics of the patients are summarized in Table 2. A total of 81 patients were analyzed. The mean patient age was 66.24 (range 42–83 y). The main presenting patterns were AUB (41 of 81 patients), a cervical smear positive for endometrial malignant cells (17 of 81 patients) and abdominal distension/discomfort (6 of 81 patients). In one case, an irregular endometrial thickening was found using TV-US. The mode of presentation was unknown in four patients. In 79 of 81 patients, the final diagnosis was SEIC/SSC, in 2 patients, it was grade 1 endometroid adenocarcinoma and in 5 cases, the SEIC was associated with intraepithelial carcinoma or serous carcinoma of the ovary. In these cases, the presence of serous ovarian carcinoma was considered as an extrauterine disease of the SEIC and it was not possible to define whether the origin of the disease was adnexal or uterine. In 31 cases, extrauterine disease, including fallopian-tube, omental, ovarian, peritoneal, bowel and lymph node metastases, focal tubal intraepithelial carcinoma and intraepithelial or serous ovarian cancer was found. All patients underwent total or radical hysterectomy, salpingo-oophorectomy and complete or partial surgical staging. A total of 66 of 81 patients did not receive any adjuvant treatment, while 12 patients underwent adjuvant chemotherapy alone, 2 patients received radiotherapy and chemotherapy and 1 patient underwent whole abdomen and pelvic radiotherapy only. In eight of the nine studies, patients with stage IA disease did not receive adjuvant therapy. In just one case, a 42-year-old patient diagnosed with SEIC stage IA received adjuvant chemotherapy [12].

### 3.3. Outcomes

All nine of the selected studies presented RR, OS, or DFS data. These results are summarized in Table 3 and Table 4. In 46 of 81 patients, there was no evidence of disease at the end of follow-up, 12 patients were alive with the disease, 18 patients died from the disease and 2 patients died from other causes. In two studies, 62.5% and 33.3% of patients receiving adjuvant chemotherapy, respectively, relapsed [2,10], while, in three studies, none of the patients relapsed [3,12,14]. Among patients not receiving any adjuvant treatment, only in one study were no relapses reported [13], and the average recurrence rate was 28.78%, ranging from 16.6% to 100%, and the DFS was 59.1%. In the patients receiving adjuvant chemotherapy, the average recurrence rate was 42.67%, ranging from 25% to 100%, and the DFS was 35.71%. In the case report by Han et al. [13], the patient developed multifocal abdominopelvic peritoneal metastases 7 years after surgery, while none of the 12 patients showed signs of disease during the average 33.1 months of follow-up. In the other studies, DFS ranged from 40% to 52.2%. The OS of the patients who underwent adjuvant chemotherapy was 57.13%, while the OS of the follow-up-only group was 66.6%.

## 4. Discussion

The scarcity of data in the literature demonstrates that SEIC is an infrequent diagnosis in clinical practice. Furthermore, from this review, it appears that 38% of cases are associated with extrauterine disease, detected at diagnosis, in contrast to the typical behavior of carcinoma in situ of the other organs. Dunton et al. detected metastases in 30–60% of SEIC cases [15]. Beyond its rarity, the understanding of this disease is also hindered by its histological characteristics. As reported in the data, it is often associated with ovarian serous carcinoma. Parallel to this other tumor, it presents a particular tropism of the peritoneal surface, even without visible locoregional infiltration. For these reasons, a diagnosis of SEIC is unlikely to be reflected in recommendations for surgical staging of endometrial carcinomas.

Two studies have described simultaneous SEIC and ovarian carcinoma. It was impossible to determine whether these were two independent neoplasms or if the SEIC had developed previously [2,14]. Serous endometrial and ovarian tumors may have similar characteristics under the microscope and may be difficult to distinguish because of their structure alone. Molecular profiling of the tumor can be helpful. This could discriminate between the presence of synchronous or metastatic tumors. Similarly, with synchronous endometrioid tumors of the endometrium and ovary, several molecular markers have been identified for differential diagnosis [16,17]. Another in-depth study that could help is the study of the tumor microenvironment, which could show differences in the genesis of both tumors [18]. Therefore, the best therapeutic management for patients with SEIC is unclear. If the tumor is clinically confined to the mucosa of the endometrium (stage IA), the most common treatment is total hysterectomy [19]. In some cases, pelvic and para-aortic lymph node removal may also be performed to assess whether the tumor has spread.

SEIC typically presents with postmenopausal bleeding, and, although the data available in the literature are numerically scarce, there is no evidence of a close correlation with risk factors such as nulliparity, late menopause, obesity and hormone replacement therapy [6]. Analysis of the literature shows that the prognosis is poor if even a minimal microscopic extrauterine disease is found, independent of the adjuvant treatment. In a study conducted by Wheeler et al., seven patients with extrauterine disease underwent chemotherapy, of which four died despite adjuvant therapy; the remaining three relapsed [2]. Conversely, the lack of extrauterine disease may be associated with a 94% chance of survival [9]. Complete surgical staging, followed by extensive sampling, is essential to define the most appropriate prognosis and therapy management. The absence of myometrial or lymphovascular space invasion was insufficient to predict the absence of extrauterine disease [20]. Due to its nature, the disease is particularly aggressive. However, in the absence of incontrovertible objective evidence of the benefit of adjuvant therapy, there is a risk of worsening iatrogenic morbidity without affecting the prognosis. Therefore, although the benefits of adjuvant treatments are not yet clear [21,22], it appears necessary to opt for additional treatments or close follow-up in patients who have undergone incomplete surgical staging. Adjuvant treatments were adopted in most cases in patients with advanced stages of the disease, except for one study in which the more aggressive approach could be justified by the young age of the patient examined, albeit with stage IA disease [12]. The patients who were followed had promising results. Of these, 19 had an extrauterine disease at diagnosis and 47 did not. However, despite being patients with stage IA disease, about one-third of patients relapsed or did not survive. A total of 66 of 81 patients had no adjuvant treatment. In particular, 20 patients died, 18 due to the disease and 2 due to other causes, while 12 patients showed signs of disease at subsequent check-ups. In most studies, it was not specified whether the disease was persistent or a recurrence.

Analyzing the data relating only to patients who did not present extrauterine disease at diagnosis, it emerged that 83% of patients subjected to FUP alone survived without evidence of disease, 6.4% developed recurrence and only approximately 1/10 of the sample died. These findings change significantly in patients with extra-uterine disease at the time of diagnosis. In fact, about half (47%) of patients undergoing follow-up alone died, 26% relapsed and the remaining percentage showed no signs of disease at subsequent follow-ups. Han described a recurrence 7 years after surgery in a woman with stage I SEIC who received an annual outpatient follow-up [13]. It is, therefore, possible to infer that follow-up is a very valid option only in patients who do not have extrauterine disease at the time of diagnosis. An additional consideration should be made regarding the incidental diagnosis of SEIC. Indeed, in clinical practice, it is more common to have a diagnosis of an intramucosal tumor following demolitive surgery. It is not always associated with adequate surgical staging. In the absence of data on the impact on the prognosis of complete staging, and given the high risk of distant microscopic involvement, this view might justify the view that adopting adjuvant therapies, even in patients with early-stage disease, could improve patient outcomes. It should be pointed out, however, that major international guidelines currently cover a limited follow-up approach [23]. However, these recommendations are based on trials that have not presented any cancer cases other than endometrioid limited to the mucosa, making their applicability questionable [24,25]. Indeed, the comparison between adjuvant and follow-up-only patients showed that adjuvant therapy could actually bring benefits in terms of overall survival and a reduction in relapses. However, if the comparison is made between patients with extrauterine disease and patients with disease confined to the uterus, examining the outcomes of patients undergoing adjuvant therapy or only follow-up in each respective group, the results partly conflict with the preliminary analysis. In particular, in patients with extrauterine disease, adjuvant therapy does not seem to have brought the expected benefits. In fact, regardless of the adjuvant therapy, about half of the patients died. That said, adjuvant treatments compared to follow-up alone have reduced rates of recurrence in patients with extrauterine disease (33.3% vs. 26.3%). In patients with disease confined to the uterus, among those subjected to only follow-up, the percentage of deaths was 10.6% while none of the patients undergoing adjuvant therapies died. However, in the latter case, the sample examined is too small from which to draw conclusions with high statistical value. Our review is severely limited by the scarcity of literature data, mainly represented by case reports. However, by reporting what has been systematically published on the topic, our review can provide a basis for further clinical knowledge concerning SEIC. In our opinion, the most important future step will be the molecular characterization of the tumor [26]. Indeed, the histological division into serous and endometroid may be limiting and unrepresentative of the molecular mechanisms underlying the curious behavior of SEIC. In particular, the most common molecular alterations in serous carcinoma of the uterus are: mutations of p53, p16 FBXW7 and PPP2R1A, HER2 overexpression, PIK3CA mutation or amplification, Cyclin E1 amplification and variable ER/PR expression [27,28]. Multiple targeted therapies have been evaluated to treat endometrial cancer in recent years. For example, dual HER2 inhibition by Trastuzumab and Pertuzumab showed antitumor activity in USC cell lines [29]. The PIK3CA inhibitor Copanlisib caused a decrease in tumor volume in five cases [30]; another preclinical result suggests that combination regimens using C-ERB/PIK3CA/AKT/mTOR inhibitors may improve responses and induce long-lasting clinical responses in patients with USC [31]; and in pre-clinical models of Cyclin E1 overexpression, the CDK2/9 inhibition has been suggested to have efficacy [32]. The routine use of the BRCA analysis was only introduced in 2014. Therefore, since the studies in question predate that date, there is a lack of data in this regard. Further studies may also consider this important prognostic factor in the therapeutic management of these patients.

In conclusion, it can be said that if the presence of SEIC is suspected, (1) it is useful to proceed to an adequate staging of the disease, in view of the high probability of extrauterine disease; (2) that the presence of an extrauterine disease significantly worsens outcomes, regardless of adjuvant treatment and that instead; and (3) in cases of disease confined to the uterine mucosa alone, the prognosis is good and follow-up allows a good control of the disease. We can, therefore, say that adjuvant therapy could further increase survival rates and reduce relapse rates in patients with uterine-confined disease and that future work, aimed at deepening the molecular signature of SEIC, will be needed to expand knowledge on this topic.

## Figures and Tables

**Table 1:** Studies’ characteristics.

Name Year	Country	Study Design	N of Participants	Surgery	Adjuvant Therapy	F/UP (months)
*Wheeler 2000* [2]	USA	Retrospective cohort-study monocentric	21	H ± BSO± Partial/complete surgical staging	None (12 pt) Platinum-based CHT (8 pt) WAPRT (1 pt)	27
*Hui* *2005* [9]	USA	Retrospective cohort-study monocentric	40	H + BSO + pelvic lymph node dissection ± omentum sampling	None	25.8
*Abushahin* *2011* [10]	USA	Case series	5	TAH+BSO± partial/complete surgical staging	None (2 pt) CHT (2 pt) RT+CHT (1 pt)	54
*Kawano* *2011* [3]	Japan	Case report	1	TAH + BSO+ EILN biopsy	6 cycles CHT (cisplatin + doxorubicin) +RT of left supraclavicolar region	37
*Pathiraja 2013* [6]	UK	Case series	5	Complete surgical staging (3 pt), incomplete surgical staging (2 pt)	None	16.6
*Ono* *2014* [11]	Japan	Prospective, cohort-study monocentric	6	RH + BSO + LN (3 pt) TAH + BSO (2 pt) TAH + BSO + OMT (1 pt)	None	36
*Kawata 2017* [12]	Japan	Case report	1	TAH + BSO + Partial OMT+ pelvic and paraaortic LN	6 cycles CHT (paclitaxel+doxorubicin+carboplatin)	9
*Han* *2020* [13]	South Korea	Case report	1	TAH + BSO + Pelvic LN	None	84
*Shimizu 2021* [14]	Japan	Case report	1	TAH + BSO + Partial OMT+ pelvic and paraaortic LN	6 cycles CHT (carboplatin, paclitaxel, bevacizumab)	9

BSO: bilateral salpingo-oophorectomy; EILN: external iliac lymph node; H: hysterectomy; LN: lymphadenectomy; OMT: omentectomy; RH: radical hysterectomy; TAH: total abdominal hysterectomy; WAPRT: whole abdomen and pelvic radiotherapy.

**Table 2:** Clinical characteristics of patients.

Name, Year	Age/ Mean Age	Presentation	Stage	Final Diagnosis	Extrauterine Disease	Follow-Up
*Wheeler, 2000* [2]	65 y	PMB (14 pt), cervical smear positive for malignant cells (6 pt), ABD (1 pt)	IA-IVB	SEIC/SSC SEIC + IC-ovary (3 pt)	IC-ovary (3 pt), fallopian tube metastases (1 pt), omentum metastases (2 pt), bowel metastases (1 pt), none (14 pt)	NED (2 pt) AWD (2 pt) DOD (3 pt) DOC (1 pt)
*Hui, 2005* [9]	66.5 y	PMB (20 pt), Cervical HPV (1 pt), pap smear positive (7 pt), ABD (8 pt), unknown (4 pt)	IA-IVB	SEIC/SSC	Omentum (14 pt) Ovary (12 pt) Fallopian tube (7 pt) Pelvic peritoneum (7 pt) Abdomen organ surface (6 pt) LN (5 pt)	NED (20 pt) DOD (9 pt) AWD (4 pt) DOC (1 pt)
*Abushahin, 2011* [10]	63.4 y	Abdominal discomfort (2 pt), PMB (1 pt), abnormal cervical smear (2 pt)	IA-IIIA	SEIC (4 pt) SEIC + OSC (1 pt)	Focal TIC (1 pt), omental implants (2 pt),	AWD (2 pt) DOD (2 pt) AWOD (1 pt)
*Kawano, 2011* [3]	61 y	PMB	IIIC	Grade 1 endometrioid adenocarcinoma	Metastatic lymph node	NED
*Pathiraja, 2013* [6]	72 y	PMB	IA	SEIC	None	NED (2 pt) DOD (2 pt) AWD (1 pt)
*Ono, 2014* [11]	74.5 y	PMB (3 pt); ABD (1 pt); abnormal cervical smear (1 pt); unknown (1 pt)	IA-IIIB	SEIC (2 pt) SSC (3 pt) SEIC-SSC (1 pt)	Omentum and ovaries micrometastases (1 pt); None (5 pt)	NED (3 pt) DOD (2 pt) AWD (1 pt)
*Kawata, 2017* [12]	42 y	AUB	IA	SEIC	None	NED
*Han, 2020* [13]	61 y	Irregular endometrial thickening observed by TV-US	IA	Grade 1 endometrioid carcinoma	None	Metastatic recurrence
*Shimizu, 2021* [14]	57 y	Abdominal pain	IIIA1 with SEIC	OSC + SEIC	Ovarian cancer, metastatic paraaortic lymph-lode	NED

ABD: abdominal distension; IC: intraepithelial carcinoma; OSC: ovarian serous cancer; PMB: postmenopausal bleeding; SSC: superficial serous carcinoma; TIC: tubal intraepithelial carcinoma; NED: no evidence of disease; AWD: alive with disease; DOD: dead of disease; DOC: dead of other causes; AWOD: alive without disease.

**Table 3:** Oncological Outcome.

Nome	3Y DFS * (%)	3Y OS ° (%)	4.5Y DFS * (%)	4.5Y OS ° (%)
*Wheeler* *2000* [2]	33.3	42.8	9.5	9.5
*Hui* *2005* [9]	17.5	17.5	10	10
*Abushahin* *2011* [10]	20	80	20	40
*Kawano* *2011* [3]	100	100	-	-
*Pathiraja* *2013* [6]	-	-	-	-
*Ono* *2014* [11]	33.3	33.3	-	-
*Kawata* *2017* [12]	-	-	-	-
*Han* *2020* [13]	100	100	100	100
*Shimizu* *2021* [14]	-	-	-	-

* Disease-Free Survival. ° Overall Survival.

**Table 4:** Recurrence Rate.

	ADJCHT			FUP		
Name	RR ’ (%)	DFS * (%)	OS °(%)	RR ’ (%)	DFS * (%)	OS °(%)
*Wheeler* *2000* [2]	62.5	25	50	0	100	91.6
*Hui* *2005* [9]	-	-	-	32.5	52.5	62.5
*Abushahin* *2011* [10]	33.3	0	33.3	50	50	100
*Kawano* *2011* [3]	0	100	100	-	-	-
*Pathiraja* *2013* [6]	-	-	-	60	40	60
*Ono* *2014* [11]	-	-	-	16.6	50	66.6
*Kawata* *2017* [12]	0	100	100	-	-	-
*Han* *2020* [13]	-	-	-	100	0	-
*Shimizu* *2021* [14]	0	100	100	-	-	-

’ Recurrence Rate * Disease-Free Survival ° Overall Survival.

## Appendix A

**Table A1:** Newcastle–Ottawa Scale.

	Selection				Comparability	Outcome			
Single-Arm Study	*Representativeness of Exposed Cohort*	*Selection of Non-Exposed Cohort*	*Ascertainment of Exposure*	*Outcome of Interest Was Not Present at Start of Study*	*Comparability of Cohorts*	*Assessment Outcome*	*Follow-Up Long Enough for Outcome to Occur?*	*Adequacy of Follow-Up*	Quality Score
*Wheeler* *2000*	+	+	+	+	+	+	-	-	6
*Hui* *2005*	-	+	+	+	-	-	-	-	3
*Abushahin* *2011*	+	+	+	+	-	+	-	-	5
*Kawano* *2011*	-	-	+	+	-	+	+	+	5
*Pathiraja* *2013*	-	+	+	+	-	+	-	-	4
*Ono* *2014*	-	+	+	+	-	+	+	+	6
*Kawata* *2017*	-	-	+	+	-	+	-	-	3
*Han* *2020*	-	-	+	+	-	+	+	+	5
*Shimizu* *2021*	-	-	+	+	-	+	-	-	3
